# Novel Hydroxy- and Epoxy-*cis*-Jasmone and Dihydrojasmone Derivatives Affect the Foraging Activity of the Peach Potato Aphid *Myzus persicae* (Sulzer) (Homoptera: Aphididae)

**DOI:** 10.3390/molecules23092362

**Published:** 2018-09-15

**Authors:** Marlena Paprocka, Anna Gliszczyńska, Katarzyna Dancewicz, Beata Gabryś

**Affiliations:** 1 Department of Botany and Ecology, University of Zielona Góra, Szafrana 1, 65-516 Zielona Góra, Poland; m.paprocka90@wp.pl (M.P.); k.dancewicz@wnb.uz.zgora.pl (K.D.); 2 Department of Chemistry, Wrocław University of Environmental and Life Sciences, Norwida 25, 50-375 Wrocław, Poland; anna.gliszczynska@wp.pl

**Keywords:** aphid probing behavior, antifeedant, structure-activity relationships

## Abstract

Jasmonates show great potential in sustainable agriculture due to their various roles in natural mechanisms of plant defense, and because they are non-toxic, non-mutagenic, and easily metabolized. The aim of the study was to explore structure–activity relationships of dihydrojasmone, *cis*-jasmone, and their derivatives at the plant–aphid interface. We focused on the behavioral responses of aphids, following the exogenous application of natural jasmonates and their derivatives to the host plants. Aphid probing behavior was examined using an electrical penetration graph technique (EPG). The chemoenzymatic transformation of *cis*-jasmone and the activity of two new derivatives are described. The application of *cis-*jasmone, dihydrojasmone, the hydroxyderivatives, epoxyderivatives, and alkyl-substituted δ-lactones hindered the foraging activity of *Myzus persicae* (Sulz.) (Hemiptera: Aphididae) during early stages of probing at the level of non-phloem tissues. The application of saturated bicyclic epoxy-δ-lactone enhanced plant acceptance by *M. persicae*. Jasmonate derivatives containing a hydroxy group, especially in correlation with a lactone ring, were more active than natural compounds and other derivatives studied. Jasmonates of the present study are worth considering as elements of sustainable aphid control as components of the “push–pull” strategy.

## 1. Introduction

Jasmonates form a family of compounds that are synthesized in plants via the octadecanoid pathway [1]. The first and the main compound in the group of jasmonates biosynthesized in this pathway is jasmonic acid (JA), which in turn serves as a basic structure for other compounds, such as methyl jasmonate, *cis*-jasmone, and jasmonic acid amino acid conjugates that are synthesized in a living cell [2,3,4]. The naturally occurring jasmonates participate in plant developmental processes as phytohormones, are involved in interactions of plants with other organisms, and are vital signaling molecules in plant responses to various abiotic and biotic stresses, including stresses caused by insect feeding [5,6,7,8].

The Food and Agriculture Organization (FAO) estimates that between 20 and 40 percent of global crop yields are reduced each year due to the damage caused by plant pests and diseases (http://www.fao.org). Arthropods destroy approximately 18–26% of annual crop production worldwide, at a value of more than $470 billion [9]. The greater proportion of losses (13–16%) occurs in the field, before harvest, and losses are the heaviest in developing countries. Pre-harvest crop loss caused by aphids may reach over 50% in maize *Zea mays* L. and 80% in wheat *Triticum aestivum* L. (Poaceae), and 80% in *Brassica* sp. (Brassicaceae) [9]. Aphids (Hemiptera: Sternorrhyncha: Aphididae) damage crops directly by removing nutrients from sieve elements and act as very efficient vectors of plant diseases. A study in Australia [10] demonstrated that feeding injury caused by *Rhopalosiphum padi* L. and *R. maidis* (Fitch) was damaging in terms of yield reduction (25.5%) and that *Barley yellow dwarf virus* transmitted by *R. padi + Sitobion miscanthi* (Takahashi) was more damaging than direct feeding, causing a yield reduction of 39% and an economic loss of $21/ha for wheat. In total, feeding and virus injuries in cereals, oilseed, and pulse crops resulted in potential economic costs of $241 and $482 million/year, respectively [10].

At present, direct control of insects in general and aphids in particular depends mainly on the use of neurotoxic insecticides. Due to the repeated applications, many aphid species, especially the peach potato aphid *Myzus persicae* (Sulzer), have developed a resistance to several aphicides [11]. It is expected that the availability and usage of conventional insecticides will be reduced due to their broad-spectrum toxicity [12] and the employment of environmentally friendly control practices will provide many benefits, including the economic ones. It is estimated that the return per dollar invested in ecologically-based biological and cultural pest controls ranges from $30 to $300, significantly higher than the $4 estimated for control based on synthetic pesticides [9]. Alternative control strategies are then required and these might include various aspects of plant signaling and volatile secondary metabolites [13]. The recognition of the crucial role of jasmonates in plant defense systems has stimulated the exploration of possibilities of practical application of these compounds in insect control for plant protection. It appeared that the exogenous application of jasmonates could affect plant metabolism to increase its potential for protection against herbivores, including those with a piercing-sucking feeding apparatus. Wheat seedlings exposed to methyl jasmonate had higher concentrations of benzoxazinoid hydroxamic acids and activity of trypsin inhibitors, which deterred *R. padi* settling and reduced the total duration of phloem sap ingestion [14]. Wheat seedlings sprayed with formulated *cis*-jasmone were less susceptible to attack by grain aphid *Sitobion avenae* (Fabricius) than control plants both in the laboratory and in the field [15]. Jasmonic acid application enhanced wheat yield by causing a reduction in the number of aphids, thrips, and wheat blossom midges [16]. Plant activators, such as *cis*-jasmone, have the potential to be adopted as a strategy to enhance a plant’s defense against herbivorous insects, as *cis*-jasmone elicits aphid-induced stress signaling in potatoes; the emission of defense volatile organic compounds (VOCs) from potatoes increases after *cis*-jasmone treatment, and plants treated with *cis*-jasmone are repellent to alate *Macrosiphum euphorbiae* (Thomas) [17]. *cis-*Jasmone treatment induced the production of defensive VOCs in field beans *Vicia faba* L. (Fabaceae), which attracted beneficial aphid parasitoids, *Aphidius ervi* Haliday (Hymenoptera: Aphidiidae) [18]. Similar effect of *cis-*jasmone application was achieved in wheat plants [19] in *Arabidopsis thaliana* L. (Brassicaceae) [20], in cotton *Gossypium hirsutum* L. (Malvaceae) [21], and in potato *Solanum tuberosum* L. (Solanaceae).

Jasmonates show great potential not only due to their various roles in natural mechanisms of plant defense and the projected practical uses but also because they are non-toxic, non-mutagenic, and easily metabolized [22]. The issues of environmental and human health effects are becoming increasingly important in the process of introducing new pesticides [23]. The use of targeted chemicals that would repel aphids or deter their probing and feeding, thus also limiting virus spread, seems a promising approach. In the course of our previous studies on aphid antifeedants, we discovered that certain modifications of the original molecule of the natural compound might lead to compounds with stronger activities or with activities of quite opposite qualities. Piperitone showed weak attractant properties and β-damascone was behaviorally inactive, but all the derived halogenolactones, and all piperitone/damascone-derived saturated and unsaturated lactones and hydroxy lactones had negative effects on the probing, feeding, and settling behavior of *M. persicae* [24,25,26,27]. A phenyl substituent at C-2 in the chromone skeleton and the introduction of an amino group at C-7 of flavone increased the aphid antifeedant activity [28]. The moderately active β-damascone ester affected aphid activities only during phloem phase, while the highly active deterrents—dihydro-β-damascol, δ-bromo-γ-lactone, and unsaturated γ-lactone—affected pre-phloem and phloem aphid probing activities [29]. We found also that the deterrent activity of decalactones against aphids depended on the size of the lactone ring and the enantiomeric purity of the compounds [30].

In our most recent studies [31,32] using various fungal strains for biotransformations, we obtained several hydroxy- and epoxy- derivatives and alkyl-substituted lactones based on the natural structures of the jasmonate group, in particular, *cis-*jasmone and dihydrojasmone. Using a simple choice test to examine their activity against the peach potato aphid, we found that the hydroxyderivative of dihydrojasmone was an aphid settling deterrent while the original compound was not, and the hydroxyderivative of *cis*-jasmone was more active than the starting compound [31]. These observations were in accordance with the results reported for hydroxyderivatives of another natural compound, β-damascone [33]. Also, in comparison to dihydrojasmone, the unsaturated lactone showed weak deterrent properties, the hydroxylactone was a weak attractant, and the saturated bicyclic epoxy-lactone was a strong attractant [32].

The aim of the present study was to explore in detail the structure–activity relationships of dihydrojasmone, *cis*-jasmone, and their derivatives at the plant–aphid interface. We focused on the behavioral responses of aphids, following the exogenous application of natural jasmonates and their derivatives to the host plants. Two new compounds and their synthesis using microbial transformations of *cis-*jasmone are described. These compounds complement the set of *cis-*jasmone and dihydrojasmone derivatives documented in the previously published works [31,32].

## 2. Results and Discussion

### 2.1. Chemical Synthesis

**Scheme 1:**
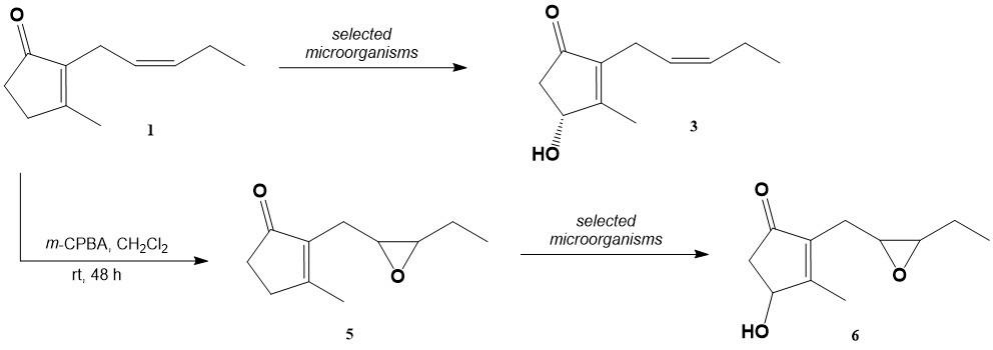
*cis*-Jasmone (**1**) and its derivatives studied towards *M. persicae*: (+)-(R)-4-hydroxyjasmone (**3**), 7,8-epoxyjasmone (**5**), and 4-hydroxy-7,8-epoxyjasmone (**6**).

The racemic (±)-7,8-epoxyjasmone (**5**) was obtained as a product of the Baeyer–Villiger reaction of *cis*-jasmone (**1**) with *m*-CPBA (Scheme 1). The structure of the obtained product (**5**) was confirmed by its spectral (IR, ^1^H, ^13^C, COSY, and HSQC NMR) data. The absorption band at 1698 cm^−1^ in the IR spectrum and the signal form carbonyl carbon atom C-1 with a chemical shift at 209.16 ppm indicated the presence of an untouched carbonyl group in this molecule. The presence of the oxiran ring in the structure of product (**5**) was proved by two signals at 2.85 and 3.03 from protons H-8 and H-7 in the ^1^H NMR spectrum, respectively. The evidence for its occurrence in the product (**5**) was provided also by ^13^C NMR, where the signals of C-7 and C-8 were moved from δ = 132.35 and δ = 125.09 for *cis*-jasmone (**1**) to δ = 55.72 and 58.66 for the product (**5**), which was the result of its connection with an oxygen atom.

In the next step of the chemoenzymatic modifications of the structure of *cis*-jasmone (**1**), we focused on the evaluation of the usefulness of wild fungal strains in obtaining the oxyderivatives of (±)-7,8-epoxyjasmone (**5**). Nine fungal strains were tested for their ability to transform the substrate (**5**). Two of them, *Absidia cylindrospora* AM336 and *Cunninghamella japonica* AM472, transformed epoxyjasmone (**5**) into the more polar metabolite **6** (Scheme 1). No formation of any biotransformation products of (±)-**5** in the cultures of another seven microorganisms was observed even after 8 days of incubation. 4-Hydroxy-7,8-epoxyjasmone (**6**) was the biotransformation product of racemic 7,8-epoxyjasmone (**5**). The structure of this hydroxyderivative was determined by its spectral data. In the ^13^C NMR spectrum of 4-hydroxy-7,8-epoxyjasmone (**6**), the chemical shift of carbon atom C-4 from 34.27 ppm in the structure of substrate to the lower field in the spectrum of product 71.64 ppm indicated the presence of an oxygen atom in this molecule. This fact was fully confirmed by a signal at 4.77 ppm from proton H-4 observed in the ^1^H NMR spectrum. The untouched structure of the oxiran ring was confirmed by two multiplets at δ = 2.87 and 3.05 from protons H-8 and H-7, respectively. Additionally, the characteristic absorption band of hydroxyl group (3428 cm^−1^) in the IR spectrum of **6** was also observed. The product of biotransformation, the 4-hydroxy-7,8-epoxyjasmone (**6**), was a new compound that has not been published before.

The most effective strain able to catalyze the conversion of epoxyjasmone (**5**) into the hydroxyderivative **6** was *Absidia cylindrospora* AM336. However, in the first day, the process of hydroxylation proceeded quite slowly and only 17% of product **6** was observed in the reaction mixture, and after two days of incubation of substrate **5** with culture *A. cylindrospora* AM336, the conversion reached 96%. In the following days, the reaction was continued until the substrate was fully consumed on the fourth day. The preparative biotransformation of (±)-**5** (100 mg) in the shaken culture of *A. cylindrospora* AM336 after 4 days gave 27 mg (25% isolated yield) of 4-hydroxy-7,8-epoxyjasmone (**6**) as a single dextrarotatory isomer (99%de).

Conversion of (±)-7,8-epoxyjasmone (**5**) by means of *Cunninghamella japonica* AM472 afforded 4-hydroxy-7,8-epoxyjasmone (**6**) in a low 4% yield but in a pure form of enantiomer (−) after 4 days of incubation.

**Scheme 2:**
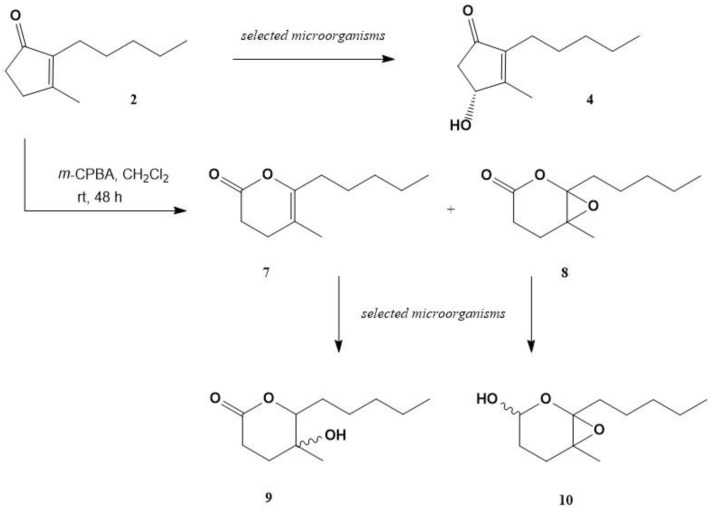
Dihydrojasmone (**2**) and its derivatives studied towards *Myzus persicae*: (+)-(*R*)-4-hydroxydihydrojasmone (**4**), 3,4-dihydro-5-methyl-6-pentyl-2*H-*pyran-2-one (**7**), 5-methyl-6-pentyl-1,13-dioxabicyclo[4.1.0]hepan-2-one (**8**), (‒)-5-hydroxy-5-methyl-6-pentyltetrahydro-2*H*-pyran-2-one (**9**), and 5-methyl-6-pentyl-1,13-dioxabicyclo[4.1.0]hepan-2-ol (**10**).

The dihydrojasmone (**2**) and its derivatives (**4**, **7**, **8**, **9**, **10**) were obtained in the course of the earlier studies [31,32] and are presented in Scheme 2.

### 2.2. Aphid Behavioral Studies

#### 2.2.1. Aphid Settling

**Figure 1:**
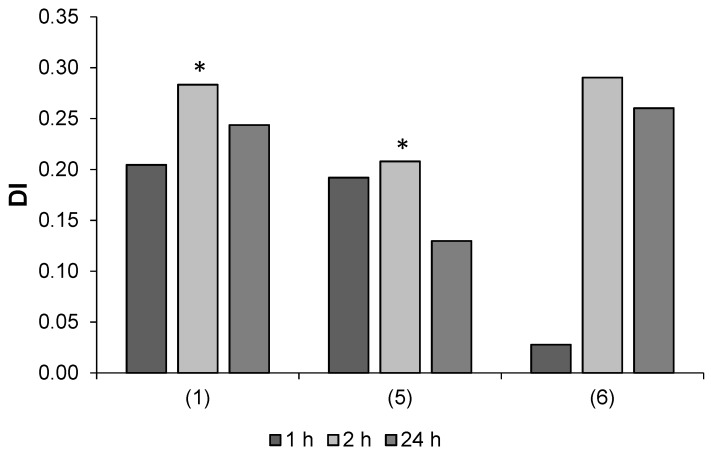
Settling success of *Myzus persicae* on *Brassica rapa* subsp. *pekinensis.* exposed to *cis*-jasmone (1), 7,8-epoxyjasmone (5), and hydroxy-7,8-epoxyjasmone (6). The same plant species was used to maintain the aphid stock culture. Relative index of deterrence (DI) after 1, 2, and 24 h. ‘*’Asterisks indicate statistically significant differences between the numbers of aphids on control and treated leaves at *p* < 0.05 (one-way ANOVA).

In the present work, we determined the settling success of the peach potato aphid after the application of the newly obtained *cis*-jasmone derivatives. The settling preferences of aphids depended on the compound and the time after the exposure of plants to the compound applied. Aphids avoided plants treated with *cis*-jasmone (**1**) and (±)-7,8-epoxyjasmone (**5**) 2 h after exposure, but the deterrent effect ceased by the 24th hour of the experiment. There was also a tendency in aphids to avoid leaves treated with 4-hydroxy-7,8-epoxyjasmone (**6**) but the trend was not significant (Figure 1). The inconstancy over time in the deterrent effect of various substances has been reported in our earlier studies, e.g., in the case of piperitone and its derivatives, and the likely explanation was that the compounds might have been degraded in the plant, translocated to other plant parts via phloem vessels, or otherwise affected by metabolic processes in the plant or the aphid [25]. 

#### 2.2.2. Aphid Behavior during Probing in Plant Tissues

The 8-h Electrical Penetration Graph (EPG) recording revealed various activities related to stylet penetration in plant tissues: “np”, non-probing, when aphid stylets were withdrawn from the plant, pathway phase “C”, when aphid stylets were inside peripheral plant tissues, epidermis and mesophyll, and phloem phase “E”, when aphid stylets were in sieve elements. Sporadically, the “F’” and “G” waveform patterns occurred indicating unidentified (“derailed”) stylet movements within plant cell walls and xylem sap uptake, respectively. Due to their rare occurrence irrespective of a treatment, the periods of “F” and “G” were included in pathway phase “C” in all calculations. Phloem phase “E” was represented by watery salivation and sap ingestion visualized as “E1” and “E2” waveform patterns, respectively. 

**Figure 2:**
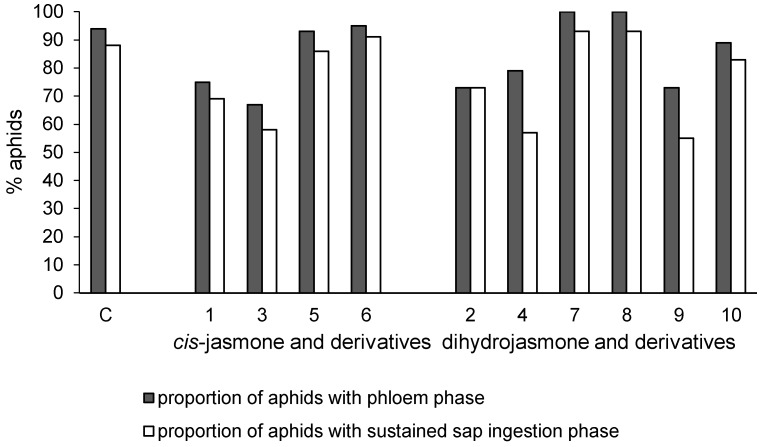
Feeding success of *Myzus persicae* on *Brassica rapa* subsp. *pekinensis* exposed to *cis-*jasmone (**1**), dihydrojasmone (**2**) and derivatives: (+)-(*R*)-4-hydroxyjasmone (**3**), (+)-(*R*)-4-hydroxydihydrojasmone (**4**), 7,8-epoxyjasmone (**5**), 4-hydroxy-7,8-epoxyjasmone (**6**), 3,4-dihydro-5-methyl-6-pentyl-2*H*-pyran-2-one (**7**), 5-methyl-6-pentyl-1,13-dioxabicyclo[4.1.0]hepan-2-one (**8**), (‒)-5-hydroxy-5-methyl-6-pentyltetrahydro-2*H*-pyran-2-one (**9**), and 5-methyl-6-pentyl-1,13-dioxabicyclo[4.1.0]heptan-2-ol (**10**). The same plant species was used to maintain the aphid stock culture.

**Figure 3:**
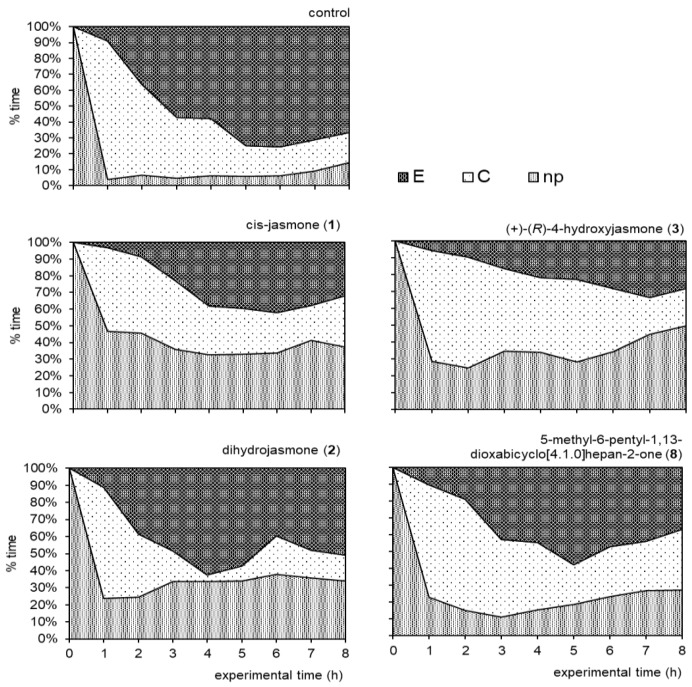
Sequential changes in *Myzus persicae* probing activity on *Brassica rapa* subsp. *pekinensis* exposed to *cis-*jasmone (**1**), dihydrojasmone (**2**), and the most active derivatives: (+)-(*R*)-4-hydroxyjasmone (**3**) (active deterrent as found in aphid settling assay by Gliszczyńska et al. [31]) and 5-methyl-6-pentyl-1,13-dioxabicyclo[4.1.0]hepan-2-one (**8**) (active attractant as found in aphid settling assay by Gliszczyńska et al. [32]). E: phloem phase (E1+E2), C: pathway phase (C+F+G), np: non-probing. The same plant species was used to maintain the aphid stock culture.

The typical behavior of *M. persicae* on the control (**C**) untreated plants consisted predominantly of active probing (93% of the experimental time), which comprised mainly of activities associated with phloem phase (60% of the probing time; phloem phase index = 0.6) (Table 1). Probing was rarely interrupted, and when it was, the non-probing intervals were relatively short: the typical probe was 1.2 h long and the typical “np” interval was 5.8 min long. The first probe was usually more than four hours long. The sieve elements were reached two hours after the onset of probing and the first period of sustained sap ingestion occurred within one hour from the first contact with the phloem. Nearly all aphids reached sieve elements and nearly all of them showed long (ca. ≤ 3-h) periods of sustained waveform E2, indicating successful feeding on treated plants. The contribution of watery salivation to the phloem phase was 10% (phloem salivation index = 0.1) (Table 1 and Table 2, Figure 2). The proportion of phloem phase increased gradually over the course of time to reach 70% of all aphid activities at the end of the experiment (Figure 3).

The present study revealed that *cis-*jasmone (**1**), dihydrojasmone (**2**), and basically all derivatives of these natural compounds (**3**–**7** and **9**–**10**) affected the course of the pre-ingestive phase of *M. persicae* probing: the first probe was significantly several times shorter than on the control plants, the probes were more numerous and separated by 2–16 min non-probing intervals, which resulted in significantly longer time when aphid stylets were outside of the plant (Table 1 and Table 2). The extended time of non-probing during the pre-phleom phase caused a trend toward the delayed commencement of feeding in sieve elements on jasmonate-treated plants. Although the significant delay was found only after the application of *cis*-jasmone (**1**), the application of the remaining jasmonates caused a failure in finding sieve elements in 20–30% of aphids during the 8-h experiment (Table 1 and Table 2). It is very likely that the proportion of aphids that failed to locate phloem would have been higher if the aphids were not tethered, which was unavoidable in the EPG experiment. Aphid reluctance to probe beyond outer layers of mesophyll may contribute to the limitation of the transmission of semi-persistent and persistent viruses [34,35]. As the aphids were visibly restless (made relatively short probes with relatively long non-probing intervals), they would have left the plants if they were allowed to move. Indeed, the free-moving aphids showed a tendency to avoid the jasmonate-treated leaves in the simple choice-assays [31,32] (and present study). Nevertheless, when the aphids did reach sieve elements, they started sap ingestion: sap consumption periods were relatively long and the contribution of watery salivation to the phloem phase was low and comparable to the control (Table 1 and Table 2). This means that no deterrent factors were detected by the aphids at the phloem level. Similar findings were reported by Cao et al. [36] who found that on methyl jasmonate-treated wheat plants, the duration of the first probe by the grain aphid *Sitobion avenae* was significantly shorter, the number of probes was significantly higher than those on control plants, and the total duration of probing was significantly shorter than on control plants. The authors suggested that the methyl jasmonate-dependent resistance factors might be due to feeding deterrents in mesophyll [36]. Indeed, the ethanolic solution of *cis-*jasmone applied to common buckwheat *Fagopyrum esculentum* (Polygonaceae) seedlings stimulated the anthocyanins accumulation [37]. *cis*-Jasmone induced changes in the defense VOCs of cotton and the VOCs from *cis*-jasmone-treated plants were significantly repellent to the cotton aphid *Aphis gossypii* Glover [21]. It is very likely that the exogenous foliar application of jasmonates in the present study put in motion plant defense mechanisms at the mesophyll level, probably the synthesis of xenobiotics, which caused the untimely termination of aphid probing. However, a plant physiology-oriented study would be necessary to reveal the phenomena beyond the reported aphid behavioral responses. The application of 5-methyl-6-pentyl-1,13-dioxabicyclo[4.1.0]hepan-2-one (**8**) had a different final effect on aphid behavior than the remaining jasmonates in the present study. Although the probes preceding the first phloem phase were relatively numerous and rather short in comparison to the control, the non-probing intervals were shorter than on the control, which resulted in the shorter time to reach phloem vessels than on the control. All aphids on (**8**)-treated leaves found sieve elements and started feeding, and sap consumption remained their main activity until the end of the experiment. The results of the EPG experiment (present study) and the settling assay [32] suggest that the application of 5-methyl-6-pentyl-1,13-dioxabicyclo[4.1.0]hepan-2-one (**8**) caused the attraction of aphids to the (**8**)-treated leaves. 

In a living cell, jasmonates undergo various modifications, such as conjugation with amino acids, glucosylation, hydroxylation, carboxylation, sulfation, and methylation, which give compounds with different biological activities. Decarboxylation of JA leads to *cis*-jasmone, which is less active than JA, but nevertheless participates in multitrophic interactions involving plants, aphids, and aphid natural enemies, hydroxylation of JA leads to less active compounds, and conjugation with amino-acids and lactonization increases the JA biological activity [8]. In the present study, the activity of natural compounds *cis*-jasmone (**1**) and dihydrojasmone (**2**) was compared to the activity of hydroxyderivatives (+)-(*R*)-4-hydroxyjasmone (**3**) and (+)-(*R*)-4-hydroxydihydrojasmone (**4**), epoxyderivatives (±)-7,8-epoxyjasmone (**5**) and 4-hydroxy-7,8-epoxyjasmone (**6**), and alkyl-substituted δ-lactones 3,4-dihydro-5-methyl-6-pentyl-2*H*-pyran-2-one (**7**), 5-methyl-6-pentyl-1,13-dioxabicyclo[4.1.0]heptan-2-one (**8**), 5-hydroxy-5-methyl-6-pentyltetrahydro-2*H*-pyran-2-one (**9**), and 5-methyl-6-pentyl-1,13-dioxabicyclo[4.1.0]heptan-2-ol (**10**) by comparing aphid behavior on leaves treated with the derivatives to aphid behavior on *cis-*jasmone- and dihydrojasmone-treated leaves (Table 1 and Table 2). The activities of hydroxyderivatives were generally similar to those of the natural compounds, with the exception of the duration of non-probing intervals, which were shorter on (**3**)-treated leaves than on *cis-*jasmone (**1**)-treated leaves and the frequency and duration of probes, which were more numerous and shorter on (**4**)-treated leaves than on dihydrojasmone (**2**)-treated leaves. The reduction in the duration of individual non-probing periods on leaves treated with (**3**) did not affect the total time that aphids spent on non-probing activities in comparison to the situation on (**1**)-treated leaves. The increase in the number of relatively short probes on (**4**)-treated leaves caused an increase in the total duration of the pathway phase activity and a slight decrease in phloem phase activities in comparison to (**2**)-treated leaves. The activity of the epoxyderivative (**5**) did not differ from the activity of *cis-*jasmone (**1**), but the application of 4-hydroxy-7,8-epoxyjasmone (**6**) caused a decrease in the duration of non-probing intervals and an increase in the duration of sap ingestion periods in comparison to the values of these parameters on *cis-*jasmone (**1**)-treated leaves. In consequence, the total non-probing time was twice as short as on *cis*-jasmone (**1**)-treated leaves and the phloem phase index was significantly higher on 4-hydroxy-7,8-epoxyjasmone (**6**)-treated leaves than on *cis-*jasmone (**1**)-treated leaves. The activities of alkyl-substituted δ-lactones that were obtained from dihydrojasmone (**2**) varied, depending on the compound. The effects of the application of 3,4-dihydro-5-methyl-6-pentyl-2*H*-pyran-2-one (**7**) on aphid behavior were similar to those caused by dihydrojasmone (**2**). The application of 5-methyl-6-pentyl-1,13-dioxabicyclo[4.1.0]hepan-2-one (**8**) and (‒)-5-hydroxy-5-methyl-6-pentyltetrahydro-2*H*-pyran-2-one (**9**) caused a significant reduction in the duration of individual non-probing intervals, but this did not affect the total time that aphids spent on non-probing activities in comparison to the effect of the dihydrojasmone (**2**) application. At the same time, the probes were more numerous and shorter on (**8**)- and (**9**)-treated leaves than on (**2**)-treated leaves, which resulted in an increase in the total duration of pathway activities and the decrease in the phloem phase index. Also, aphids on (**8**)-treated leaves were more successful in finding sieve elements and feeding than the aphids on (**2**)-treated leaves. The application of 5-methyl-6-pentyl-1,13-dioxabicyclo[4.1.0]heptan-2-ol (**10**) caused only slight changes in aphid behavior as compared to the dihydrojasmone (**2**) treatment, with no significant effect on the overall behavior of aphids (Table 1 and Table 2). 

All jasmonates that were the subject of the present study are worth considering as elements of sustainable aphid control, possibly as components of the “push–pull” strategy. In this approach to insect control, pests are repelled or deterred from the crop (the “push”) and simultaneously attracted (the “pull”) to other areas, such as trap crops or barrier crops, which act as “sinks” for non-persistent viruses (viruses are lost by infective vectors while probing on plants of barrier crops) or mechanical obstacles that impede the colonization of the protected crop [38,39]. The reluctance to probe beyond outer layers of mesophyll may contribute to the limitation of the transmission of semi-persistent and persistent viruses [34,35]. Considering the results of the present study, 5-methyl-6-pentyl-1,13-dioxabicyclo[4.1.0]heptan-2-one (**8**), an attractant that enhances the process of plant acceptance by *M. persicae*, may be used as a “pull” component, while all the remaining substances, the deterrents that impede the ability of the peach potato aphid to recognize the host plant during the early stages of probing, may be applied as a “push” component.

## 3. Material and Methods

### 3.1. Compounds

The structures of compounds **1**–**10** applied for the probing and feeding behavior experiments on *M. persicae* are presented in Scheme 1 and Scheme 2. *cis*-Jasmone (**1**) and dihydrojasmone (**2**) were purchased from Sigma Aldrich (Sigma Aldrich, St. Louis, MI, USA) (*cis*-jasmone W319600 Sigma Aldrich), dihydrojasmone W376302 Sigma Aldrich). The hydroxyderivatives **3** and **4** were obtained in the process of microbial transformation of *cis*-jasmone (**1**) and dihydrojasmone (**2**), respectively, which was the content of the paper reported before [31]. Epoxyjasmone (**5**) and alkyl-substituted δ-lactones (**7**) and (**8**) were synthesized in the reaction with *m-*chloroperoxybenzoic acid and were subsequently converted into oxyderivatives **6**, **9**, and **10** using the enzymatic system of wild fungal strains [32].

#### 3.1.1. Microorganisms

In the process of transformation of racemic 7,8-epoxyjasmone (**5**), nine wild fungal strains (*Fusarium coulmorum* AM10, *Fusarium equiseti* AM15, *Botrytis cinerea* AM235, *Absidia glauca* AM254, *Bauveria bassiana* AM278, *Absidia cylindrospora* AM336, *Cunninghamella japonica* AM472, *Chaetomium* sp. KCh6651, and *Didymosphaeria igniaria* KCh6670) that came from the Department of Chemistry, Wroclaw University of Environmental and Life Sciences (Wroclaw, Poland) were tested as biocatalysts. The aim of using the wild type of fungal strains in the process of biotransformation was the economy aspect of the performed experiments and processes, the results of which can potentially be applied in the industry. Microorganisms were maintained on Sabouraud 4% dextrose-agar slopes at 4 °C and freshly subcultured before use in the transformation experiments.

All screening experiments, as well as the biotransformations of (±)-7,8-epoxyjasmone (**5**) in the preparative scale, were carried out according to the method described earlier [31]. A portion of 1 mL of the pre-incubation culture solution was used to inoculate two or three 2000 mL flasks, each containing 500 mL of the cultivation medium. The cultures were inoculated at 25 °C for 48–72 h on a rotary shaker (150 rpm). Then, 50 mg of a substrate was dissolved in 5 mL of acetone added to each flask. After 1–6 days (depending on the culture) of incubation, the mixtures were extracted with dichloromethane (3 × 300 mL), dried (MgSO4) and concentrated in vacuo. The transformation products were separated using column chromatography [32].

#### 3.1.2. Analysis

Gas chromatography (GC) analysis was carried out using a HP-5 column (cross-linked methyl silicone gum, 30 m × 0.32 mm × 0.25 μm) and an FID detector using the following temperature program: injector 250 °C, detector (FID) 250 °C, column temperature: 70 °C, 70–175 °C (rate 20 °C/min), 175–300 °C (rate 40 °C/min), 300 °C (hold 2 min). The enantiomeric compositions of the obtained products were determined using chiral GC analysis with a CP-cyclodextrin-B (25 m × 0.25 mm × 0.25 μm) chiral column. The temperature program was as follows: 80 °C, 80–200 °C (rate 0.6°/min), 200 °C (hold 1 min). NMR spectroscopic measurements were carried out for CDCl_3_ solutions on a Bruker Avance AMX 300 spectrometer (Bruker, Rheinstetten, Germany). IR spectra were performed for liquid films with Thermo-Nicolet IR300 spectrophotometer. Optical rotations were measured on JASCO P-2000-Na digital polarimeter (JASCO Inc., Easton, MD, USA) with iRM controller using dichloromethane as a solvent (concentration denoted in g/100 mL). 

*(±)-7,8-Epoxyjasmone (**5**)*: oily liquid, ^1^H NMR (CDCl_3_), δ: 1.04 (t, *J* = 7.5 Hz, 3H, CH_3_-10), 1.61 (m, 2H, CH_2_-9), 2.26 (s, 3H, CH_3_-11), 2.27 (dd, *J* = 14.3 and 7.3Hz, 1H, one of CH_2_-6), 2.39 (m, 2H, CH_2_-4), 2.51-2.54 (m, 3H, CH_2_-5 and one of CH_2_-6), 2.85 (m, 1H, H-8), 3.03 (m, 1H, H-7); ^13^C NMR (CDCl_3_), δ: 209.16 (C-1), 172.55 (C-3), 136.85 (C-2), 58.66 (C-8), 55.72 (C-7), 34.27 (C-4), 31.89 (C-5), 22.09 (C-6), 21.24 (C-9), 17.59 (C-11), 10.64 (C-10); IR (film, cm^−1^): 1698 (s), 1648 (s), 1385 (m).

*4-Hydroxy-7,8-epoxyjasmone (6)*: oily liquid, ^1^H NMR (CDCl_3_), δ: 1.06 (t, *J* = 7.5 Hz, 3H, CH_3_-10), 1.63 (m, 2H, CH_2_-9), 1.72 (s, 1H, -OH), 2.14 (s, 3H, CH_3_-11), 2.27–2.33 (m, 2H, one of CH_2_-5 and one of CH_2_-6), 2.54–2.57 (m, 1H, one of CH_2_-6), 2.82 (dd, 1H, *J* = 718.4 and 6.12 Hz one of CH_2_-5), 2.87 (m, 1H, H-8), 3.05 (m, 1H, H-7), 4.77 (m, 1H, H-4). ^13^C NMR (CDCl_3_), δ: 204.87 (C-1), 170.61 (C-3), 138.33 (C-2), 71.64 (C-4), 58.60 (C-8), 55.31 (C-7), 44.08 (C-5), 21.97 (C-6), 21.12 (C-9), 13.91 (C-11), 10.53 (C-10). IR (film, cm^−1^): 3428 (s), 1702 (s), 1649 (s).

### 3.2. Aphid Behavioral Studies

#### 3.2.1. Cultures of Aphids and Plants

The peach potato aphids and the Chinese cabbage *Brassica rapa* subsp. *pekinensis* (Lour.) Hanelt were reared in the laboratory at 20 °C, 65% r.h., and L16:8D photoperiod. One to seven days old apterous females of *M. persicae* and 3-week old plants with four to five fully developed leaves were used for experiments. *M. persicae* were obtained from the laboratory culture maintained at the Department of Botany and Ecology for many generations since 2008. All experiments were carried out under the same conditions of temperature, relative humidity, and photoperiod. The bioassays were started at 10:00–11:00 a.m.

#### 3.2.2. Application of Compounds

Each compound was dissolved in 70% ethanol to obtain the recommended 0.1% solution [40]. All compounds were applied on the adaxial and abaxial leaf surfaces by immersing a leaf in the ethanolic solution of a given compound for 30 s [26,29]. Control leaves of similar size were immersed in 70% ethanol that was used as a solvent for the studied jasmonates. Experiments were performed 1 h after the compound’s application to allow for the evaporation of the solvent. Every plant and aphid were used only once.

#### 3.2.3. Aphid Settling

This bioassay allows the study of aphid host preferences under semi-natural conditions. Aphids were given free choice between control and treated excised leaves that were placed in a Petri dish. Aphids were placed in the dish equidistance from treated and untreated leaves, so that aphids could choose between treated (on one half of a Petri dish) and control leaves (on the other half of the dish). Aphids that settled, i.e., they did not move and the position of their antennae indicated feeding [41], on each leaf were counted at 1 h, 2 h, and 24 h intervals after access to the leaf. Each experiment was replicated eight times (eight replicates, 20 viviparous apterous females/replicate). Aphids that were moving or not on any of the leaves were not counted. The data were analyzed using a Student’s *t*-test (STATISTICA 6.1. package). If aphids showed a clear preference for the leaf treated with the tested compound (*p* < 0.05), the compound was described as having attractant properties. If aphids settled mainly on the control leaf (*p* < 0.05), the compound tested in the respective choice test was stated as being a deterrent. From the data thus obtained, the relative index of deterrence (DI) was calculated: DI = (C-T/C+T) where C was the number of aphids settled on control leaf, T was the number of aphids settled on the leaf treated with tested compound. The value of DI ranged between 1 (ideal deterrent) and −1 (ideal attractant). 

#### 3.2.4. Aphid Probing Behavior

Probing by *M. persicae* in tissues of plants treated with jasmonates was monitored using the technique of electronic registration of aphid behavior known as an EPG (electrical penetration graph) that is frequently employed in insect–plant relationship studies [42,43]. By using the EPG technique, it is possible to monitor aphid probing and feeding behavior within plant tissues, localize natural plant resistance factors, and reveal the effect of exogenously applied compounds that may influence plant–aphid interactions [28,36,44,45]. Aphid decisions whether to leave or remain on the plant and continue probing and feeding are based mainly on chemical information within plant tissues that is detected with contact chemoreceptors in the gustatory organ in the pharynx [46]. At the same time, the alteration of aphid behavior during probing following the exogenous application of xenobiotics demonstrates indirectly the transcuticular and within-plant transfer of these xenobiotics [44]. The EPG technique allows a separate analysis of aphid behavior at pre-ingestive (within non-phloem tissues before the first phloem phase) and ingestive (within the phloem) phases of probing [46]. In the experimental set-up, aphid and plant are made parts of an electrical circuit, which is completed when the aphid inserts its stylets into the plant. A weak voltage is supplied in the circuit, and all changing electric properties are recorded as EPG waveforms that can be correlated with aphid activities and stylet position in plant tissues [47]. The parameters describing aphid behavior during probing and feeding, such as the total time of probing, proportion of phloem patterns, number of probes, etc., are good indicators of plant suitability or interference of probing by chemical or physical factors in plant tissues [48]. Jasmonates were applied to test leaves of intact plants as described earlier. Aphids were attached to a golden wire electrode with silver paint and starved for 1 h prior to the experiment. The experiments were repeated 25 times for each jasmonate/aphid combination and each replicate consisted of a freshly prepared aphid and a plant. Incomplete recordings were rejected from analysis. The probing behavior of aphids was monitored for 8 h continuously. A Giga-8 DC EPG system with a 1 GΩ of input resistance (EPG Systems, Wageningen, The Netherlands) was used to record the EPG waveforms. EPGs were recorded and analyzed using Stylet+ Software (EPG Systems, Wageningen, The Netherlands). Signals were saved on the computer and the various behavioral phases were labelled manually using the Stylet+ software. The following EPG patterns were distinguished: np (non-penetration, when aphid stylets did not have contact with plant tissues), A, B, C, F (pathway phase—penetration of non-phloem tissues), E1 (salivation into sieve elements), E2 (ingestion of phloem sap), and G (ingestion of xylem sap). The E1/E2 transition patterns were included in E2. EPG parameters were calculated manually and individually for every aphid. The waveform patterns that were terminated by the end of the experimental period (8 h) were not excluded from the calculations. The relevant information is provided in the explanation to the tables. The mean and standard errors were subsequently calculated using the EPG analysis Excel worksheet created for this study. The experimental results were analyzed with a one-tailed Mann–Whitney U-test using the STATISTICA 9.0 package (StatSoft, Tulsa, OK, USA).

## 4. Conclusions

The behavioral study demonstrated that *cis-*jasmone (**1**), dihydrojasmone (**2**), and the hydroxyderivatives: (+)-(*R*)-4-hydroxyjasmone (**3**) and (+)-(*R*)-4-hydroxydihydrojasmone (**4**), epoxyderivatives: (±)-7,8-epoxyjasmone (**5**) and 4-hydroxy-7,8-epoxyjasmone (**6**), and alkyl-substituted δ-lactones: 3,4-dihydro-5-methyl-6-pentyl-2*H*-pyran-2-one (**7**), 5-hydroxy-5-methyl-6-pentyltetrahydro-2*H*-pyran-2-one (**9**), and 5-methyl-6-pentyl-1,13-dioxabicyclo[4.1.0]heptan-2-ol (**10**) impeded aphid activity during early stages of probing, at the level of non-phloem tissues, which caused a failure in finding the food source in plant sieve elements. 5-Methyl-6-pentyl-1,13-dioxabicyclo[4.1.0]heptan-2-one (**8**) enhanced the process of plant acceptance by *M. persicae*. The chemical structure of the studied compounds had a significant influence on their effect on aphid behavior. The presence of a hydroxy group in the structure of jasmonates played a key role in this respect, in particular when it was correlated with the lactone ring. Jasmonate derivatives containing these two structural fragments were more active biologically than the other compounds studied.

## Figures and Tables

**Table 1:** Probing behavior (EPG parameters) of *Myzus persicae* on *Brassica rapa* subsp. *pekinensis* leaves treated with *cis*-jasmone (**1**) and its derivatives: (+)-(*R*)-4-hydroxyjasmone (**3**), 7,8-epoxyjasmone (**5**), and 4-hydroxy-7,8-epoxyjasmone (**6**) ^A^. The same plant species was used to maintain the aphid stock culture.

EPG Parameter/Compound	C	1	3	5	6
**General Aspects of Aphid Probing Behavior**					
	*n* = 17	*n* = 16	*n* = 12	*n* = 14	*n* = 22
Total duration of np ^a^ (h)	0.6 ± 0.3	3.1 ± 0.6 *	2.8 ± 0.7 *	1.5 ± 0.6 *	1.2 ± 0.3 *^,†^
Total duration of pathway (h)	2.9 ± 0.5	2.7 ± 0.3	3.6 ± 0.6	3.0 ± 0.4	3.2 ± 0.4
Total duration of phloem phase (h)	4.5 ± 0.6	2.2 ± 0.5 *	1.7 ± 0.6 *	3.5 ± 0.6	3.5 ± 0.5
Phloem phase index ^b^	0.6 ± 0.1	0.4 ± 0.1 *	0.3 ± 0.1 *	0.5 ± 0.1	0.5 ± 0.1 ^†^
Number of probes	6.4 ± 2.0	21.4 ± 3.0 *	34.3 ± 7.0 *	19.9 ± 3.9 *	22.0 ± 4.1 *
Mean duration of a probe (min)	70.8 ± 13.9	13.2 ± 2.4 *	9.1 ± 1.7 *	19.6 ± 3.3 *	18.4 ± 2.8 *
Mean duration of np intervals ^c^	5.8 ± 1.5	7.2 ± 1.8	4.6 ± 1.4 *^,†^	4.7 ± 2.0	3.4 ± 0.5 ^†^
**Activities in Non-Phloem Tissues before the First Phloem Phase**					
	*n* = 17	*n* = 16	*n* = 12	*n* = 14	*n* = 22
Number of probes	2.1 ± 1.0	8.8 ± 2.6 *	20.1 ± 5.0 *	4.7 ± 0.8 *	10.5 ± 2.5 *
Duration of first probe (min)	249.1 ± 54.5	10.6 ± 5.1 *	8.1 ± 4.0 *	29.9 ± 16.5 *	10.3 ± 4.9 *
Time from first probe to first phloem phase(h)	2.0 ± 0.5	3.5 ± 0.7	4.0 ± 0.9	1.6 ± 0.5	2.1 ± 0.4
Time from first probe to first sustained sap ingestion phase E2 > 10 min (h)	2.8 ± 0.6	4.6 ± 0.6 *	4.8 ± 0.9	3.1 ± 0.7	2.5 ± 0.5 ^†^
Total duration of np (min) ^c^	8.5 ± 3.6	107.5 ± 40.6 *	101.4 ± 46.3 *	36.5 ± 27.7	26.3 ± 8.5 *
Mean duration of np intervals (min)^c^	4.0 ± 1.6	11.0 ± 3.6	4.8 ± 1.7 *^,†^	6.6 ± 4.9	2.5 ± 0.5
**Activities in Sieve Elements ^B^**					
Duration of first phloem phase (min)	160.2 ± 47.6	60.8 ± 31.9	57.8 ± 44.7	100.7 ± 41.4	149.8 ± 33.0 ^†^
	*n* = 16	*n* = 12	*n* = 8	*n* = 13	*n* = 21
Duration of first sap ingestion phase (min)	159.7 ± 47.6	63.3 ± 31.4	92.2 ± 51.6	110.5 ± 43.7	156.5 ± 33.7 ^†^
	*n* = 16	*n* = 12	*n* = 8	*n* = 12	*n* = 20
Mean duration of sap ingestion (min)	175.8 ± 44.9	81.0 ± 30.0	101.0 ± 50.3	107.9 ± 39.7	162.7 ± 31.7
	*n* = 16	*n* = 12	*n* = 8	*n* = 12	*n* = 20
Phloem salivation index ^d^	0.1 ± 0.0	0.04 ± 0.01	0.1 ± 0.0 *	0.1 ± 0.1	0.1 ± 0.1
	*n* = 16	*n* = 12	*n* = 8	*n* = 13	*n* = 21

^A^ Values are means ± SE; *n* = number of replications; *^,†^—significant differrence in relation to control (**C**) and *cis-*jasmone (**1**), respectively (*p* < 0.05, Mann–Whitney U test); ^B^ only aphids that showed a phloem phase were used for analysis; ^a^ np = non-probing; ^b^ E1 + E2/C + E1 + E2 + G + F; ^c^ excluding the first non-probing period; ^d^ E1/E1 + E2.

**Table 2:** Probing behaviour (EPG parameters) of *Myzus persicae* on *Brassica rapa* subsp. *pekinensis* leaves treated with dihydrojasmone (**2**) and its derivatives: (+)-(*R*)-4-hydroxydihydrojasmone (**4**), 3,4-dihydro-5-methyl-6-pentyl-2*H*-pyran-2-one (**7**), 5-methyl-6-pentyl-1,13-dioxabicyclo[4.1.0]hepan-2-one (**8**), (‒)-5-hydroxy-5-methyl-6-pentyltetrahydro-2*H*-pyran-2-one (**9**), and 5-methyl-6-pentyl-1,13-dioxabicyclo[4.1.0]hepan-2-ol (**10**)^A^. The same plant species was used to maintain the aphid stock culture.

EPG Parameter/Compound	C	2	4	7	8	9	10
**General aspects of aphid probing behaviour**							
	*n* = 17	*n* = 15	*n* = 14	*n* = 14	*n* = 15	*n* = 11	*n* = 18
Total duration of np ^a^ (h)	0.6 ± 0.3	2.6 ± 0.8 *	1.5 ± 0.4 *	1.6 ± 0.4 *	1.6 ± 0.4 *	2.0 ± 0.8 *	1.3 ± 0.5 *
Total duration of pathway (h)	2.9 ± 0.5	1.8 ± 0.4	3.9 ± 0.7 ^†^	2.8 ± 0.5	3.4 ± 0.4 ^†^	3.5 ± 0.7	2.8 ± 0.5
Total duration of phloem phase (h)	4.5 ± 0.6	3.6 ± 0.7	2.7 ± 0.8	3.7 ± 0.6	3.0 ± 0.6	2.4 ± 0.9	3.9 ± 0.7
Phloem phase index ^b^	0.6 ± 0.1	0.5 ± 0.1	0.4 ± 0.1	0.6 ± 0.1	0.4 ± 0.1	0.3 ± 0.1	0.5 ± 0.1
Number of probes	6.4 ± 2.0	12.3 ± 2.8 *	23.4 ± 4.2 *^,†^	16.5 ± 3.3 *	28.0 ± 6.2 *^,†^	26.0 ± 5.2 *^,†^	19.1 ± 3.6 *
Mean duration of a probe (min)	70.8 ± 13.9	26.5 ± 5.7 *	16.7 ± 3.1 *^,†^	23.3 ± 4.4 *	13.7 ± 2.3 *^,†^	13.8 ± 2.8 *^,†^	20.9 ± 3.9 *^,†^
Mean duration of np intervals ^c^	5.8 ± 1.5	13.2 ± 4.7	3.9 ± 0.8	4.6 ± 1.4	3.1 ± 0.8 *^,†^	3.7 ± 1.6 *^,†^	3.7 ± 1.0
**Activities in non-phloem tissues before 1st phloem phase**							
	*n* = 17	*n* = 15	*n* = 14	*n* = 14	*n* = 15	*n* = 11	*n* = 18
Number of probes	2.1 ± 1.0	7.1 ± 2.5 *	14.7 ± 3.4 *	7.2 ± 1.6 *	6.5 ± 1.5 *	14.0 ± 4.6 *	10.3 ± 2.6 *
Duration of first probe (min)	249.1 ± 54.5	37.4 ± 25.9 *	34.6 ± 30.0 *	8.3 ± 5.7 *	16.9 ± 8.2 *	5.6 ± 2.7 *	66.7 ± 36.1 *
Time from first probe to first phloem phase(h)	2.0 ± 0.5	2.9 ± 0.8	3.6 ± 0.8	1.6 ± 0.3	1.4 ± 0.2	3.2 ± 1.0	2.6 ± 0.6
Time from first probe to first sustained sap ingestion phase E2> 10 min (h)	2.8 ± 0.6	3.3 ± 0.8	3.8 ± 1.0	2.7 ± 0.6	2.4 ± 0.5	5.0 ± 1.0	3.0 ± 0.7
Total duration of np (min) ^c^	8.5 ± 3.6	119.1 ± 49.6 *	69.2 ± 27.7 *	17.5 ± 4.9 *	13.2 ± 6.0	56.0 ± 36.4 *	44.2 ± 24.0 *
Mean duration of np intervals (min) ^c^	4.0 ± 1.6	16.7 ± 6.8	4.8 ± 1.2	2.4 ± 0.4	2.0 ± 0.7 *^,†^	4.0 ± 2.6 *^,†^	4.3 ± 1.8 *^,†^
**Activities in sieve elements ^A^**							
Duration of first phloem phase (min)	160.2 ± 47.6	124.4 ± 49.5	131.0 ± 53.0	160.5 ± 42.8	38.7 ± 17.1	80.3 ± 53.1	211.6 ± 50.0
	*n* = 16	*n* = 11	*n* = 11	*n* = 14	*n* = 15	*n* = 8	*n* = 16
Duration of first sap ingestion phase (min)	159.7 ± 47.6	161.2 ± 53.3	132.8 ± 58.5	170.6 ± 44.7	60.0 ± 24.8	108.4 ± 58.2	211.4 ± 49.8
	*n* = 16	*n* = 11	*n* = 10	*n* = 13	*n* = 15	*n* = 7	*n* = 16
Mean duration of sap ingestion (min)	175.8 ± 44.9	167.6 ± 45.0	151.5 ± 55.7	164.3 ± 45.3	68.2 ± 22.3 *	135.8 ± 56.4	224.7 ± 47.2
	*n* = 16	*n* = 11	*n* = 10	*n* = 13	*n* = 15	*n* = 7	*n* = 16
Phloem salivation index ^d^	0.1 ± 0.0	0.01 ± 0.00	0.2 ± 0.1	0.1 ± 0.1	0.1 ± 0.0 ^†^	0.2 ± 0.1	0.03 ± 0.01
	*n* = 16	*n* = 11	*n* = 11	*n* = 14	*n* = 15	*n* = 8	*n* = 17

^A^ Values are means ± SE; *n* = number of replications; *^,†^—significant difference in relation to control (**C**) and dihydrojasmone (**2**), respectively (*p* < 0.05, Mann–Whitney U test); ^B^ only aphids that showed a phloem phase were used for analysis; ^a^ np = non-probing; ^b^ E1 + E2/C + E1 + E2 + G + F; ^c^ excluding the first non-probing period; ^d^ E1/E1 + E.
